# Correlations between DNA methylation levels and nucleosome enrichment in the human genome

**DOI:** 10.1186/1756-8935-6-S1-O9

**Published:** 2013-03-18

**Authors:** Clayton K Collings, John N Anderson

**Affiliations:** 1 Department of Biological Sciences, Purdue University, West Lafayette, IN, 47907, USA

## 

Next generation sequencing studies have contributed further evidence that the methylation of CpG dinucleotides can alter protein-DNA interactions, and this epigenetic mechanism is thought to play a critical role in the control of gene expression in higher eukaryotic organisms. Recent analyses of high resolution nucleosome maps have revealed novel relationships between DNA methylation and localization of nucleosomes [1,2]. In our latest work, we conducted high-throughput nucleosome reconstitution experiments on 572 KB of human DNA that was unmethylated or methylated by bacterial CpG methyltransferase in order to investigate the effects of this epigenetic modification on the positioning and stability of nucleosomes *in vitro*[2]. The results demonstrated that a subset of nucleosomes positioned by nucleotide sequence was sensitive to methylation where the modification increased the affinity of these sequences for the histone octamer. The features that distinguished these nucleosomes from the bulk of the methylation-insensitive nucleosomes were an increase in the frequency of CpG dinucleotides and a unique rotational orientation of CpGs such that their minor grooves tended to face toward the histones in the nucleosome rather than away. These methylation-sensitive nucleosomes were preferentially associated with exons as compared to introns while unmethylated CpG islands near transcription start sites became enriched in nucleosomes upon methylation. In order to provide additional validation for these findings, we examined the influence of CpG methylation levels [3] on the genome-wide distributions of nucleosomes reconstituted *in vitro *[4] derived from leukocyte DNA. Within DNA sequence contexts of high G+C and CpG content, nucleosome occupancy was found not only to be enriched but also strongly correlated with levels of DNA methylation, and these optimal sequence environments were particularly accommodated by exons and CpG islands over other genomic components. Additionally, throughout the predominantly methylated genome, the minor grooves of methylated CpG dinucleotides favoured to lie inwards relative to the histone surface while unmethylated CpGs displayed no rotational orientation. The results in this report support our previous proposal, suggesting that DNA methylation is more directly involved in dictating nucleosome organization, especially in exons and CpG islands. Current efforts entailing comparisons of nucleosome assembly in cells to methylomes are underway to evaluate the effects of DNA methylation on the positioning and stability of nucleosomes *in vivo*.
